# Supplementary material for: Parallel diversifications of Cremastosperma and Mosannona (Annonaceae), tropical rainforest trees tracking Neogene upheaval of South America
Source: R Soc Open Sci. 2018 Jan 31;5(1):171561. doi: 10.1098/rsos.171561 (PMC5792937; doi:10.1098/rsos.171561)
Supplement: Accessions details [file rsos171561supp1.pdf]

Appendix 1: Accession details for samples used in phylogenetic analyses, with GenBank accession numbers. Malate synthase was sequenced in two non-overlapping parts that have been given separate GenBank accession numbers.

Species	Collection	Country	*matK*	*ndhF*	*rbcL*	*atpB-rbcL*	*psbA-trnH*	*trnTL*	*trnLF*	pseud*trnLF*	malate synthase	PHYC
Annickia pilosa (Exell) Setten & Maas	Sosef 1803 (WAG)	Gabon	AY743488	AY841402	AY743450	AY841371	AY841444	AY841572	AY743469	—	—	—
Klarobelia inundata Chatrou	Chatrou 205 (U)	Peru	AY743490	AY841409	AY743452	AY841378	AY841469	AY841577	AY743471	—	—	—
Klarobelia stipitata Chatrou	Chatrou 113 (U)	Costa Rica	—	—	AY841628	—	AY841472	—	AY841706	—	—	—
Ephedranthus parviflorus S.Moore	Prance 19246 (U)	Brazil	—	—	AY841615	—	AY841462	—	AY841693	—	—	MG680677
Ephedranthus sp.	Maas 8826 (U)	Brazil	AY841396	AY841407	AY841616	AY841376	AY841463	AY841575	AY841694	—	—	—
Malmea dielsiana R.E.Fr.	Chatrou 122 (U)	Peru	AY238964	AY841410	AY238955	AY841379	AY841473	AY841578	AY231288 AY238948	DQ018195	—	MG680678
Malmea sp.	Chatrou 8 (U)	Peru	AY841397	AY841411	AY841527	AY841380	AY841475	AY841579	AY841541	DQ018196	—	—
Onychopetalum periquino (Rusby) D.M.Johnson & N.A. Murray	Chatrou 425 (U)	Bolivia	AY518876	AY841414	AY319065	AY841383	AY841485	AY841582	AY319179	—	—	—
Oxandra macrophylla R.E.Fr.	Chatrou 204 (U)	Peru	—	—	AY841642	—	AY841491	—	AY841720	—	—	MG680679
Oxandra venezuelana R.E.Fr.	Chatrou 120 (U)	Costa Rica	—	—	AY841645	—	AY841495	—	AY841723	—	—	MG680680
Pseudomalmea diclina (R.E.Fr.) Chatrou	Chatrou 211 (U)	Peru	AY841398	AY841419	AY319068	AY841388	AY841506	—	AY319128	—	MG680649 MG680658	MG680681
Pseudoxandra lucida R.E.Fr.	Chatrou 212/213 (U)	Peru	AY518870	AY841420	AY319076	AY841389	AY841510	AY841588	AY319190	—	—	—
Pseudoxandra spiritus-sancti Maas	Maas 8833 (U)	Brazil	AY841399	AY841421	AY841533	AY841390	AY841513	AY841589	AY841547	—	—	—

Species	Collection	Country	*matK*	*ndhF*	*rbcL*	*atpB-rbcL*	*psbA-trnH*	*trnTL*	*trnLF*	pseud*trnLF*	malate synthase	PHYC
Sapranthus viridiflorus G.E.Schatz	Chatrou 55 (U)	Costa Rica	AY743493	AY841422	AY319051	AY841391	AY841515	AY841590	AY319165	—	—	—
Unknown genus	Tello 3416 (NY)	Peru	AY841520	—	—	—	AY841456	—	AY841534	—	—	—
Cremastosperma brevipes (DC) R.E.Fr.	Scharf 76 (U)	French Guiana	AY743550	AY841405	AY743527	AY841374	AY841447	AY841573	AY743573	DQ018191	—	MG680676
Cremastosperma bullatum Pirie	Pirie 71 (U)	Peru	AY743560	DQ018140	AY743537	—	AY841459	DQ018170	AY743583	DQ018220	—	—
Cremastosperma bullatum Pirie	Pirie 94 (U)	Peru	DQ018276	DQ018142	DQ018235	—	DQ018256	DQ018172	DQ018188	DQ018221	—	—
Cremastosperma cauliflorum R.E.Fr.	Maas 9029 (U)	Peru	AY743548	DQ018125	AY743525	—	DQ018240	DQ018150	AY743571	DQ018206	—	—
Cremastosperma cauliflorum R.E.Fr.	Chatrou 224 (U)	Peru	AY743542	AY841406	AY743519	AY841375	AY841448	AY841574	AY743565	DQ018192	—	—
Cremastosperma gracilipes R.E.Fr.	Chatrou 267 (U)	Ecuador	AY743544	—	AY743521	—	—	—	AY743567	—	—	—
Cremastosperma leiophyllum (Diels) R.E.Fr.	Pirie 2 (U)	Bolivia	AY743546	DQ018123	AY743523	—	AY841449	DQ018148	AY743569	DQ018193	—	—
Cremastosperma longicuspe R.E.Fr.	Woytkowski 7128 (GH, MO, UC, US)	Peru	MG680703	MG680667	MG649385	—	MG680710	MG649386	MG680725	MG649387	—	—
Cremastosperma monospermum (Rusby) R.E.Fr.	Killip 28691 (US, F, NY)	Peru	AY743566	—	AY743533	—	—	—	AY743579	—	—	—
Cremastosperma macrocarpum Maas	Wingfield 6751 (U)	Venezuela	AY743551	DQ018129	AY743528	—	AY841450	DQ018154	AY743574	DQ018194	—	—
Cremastosperma magdalenae Pirie	Escobar 3309 (U)	Colombia	DQ018279	DQ018143	AY841521	—	AY841460	DQ018174	AY841535	—	—	—
Cremastosperma megalophyllum	Maas 8595 (U)	Ecuador	AY743540	—	AY743517	—	—	—	AY743563	—	—	—

Species	Collection	Country	*matK*	*ndhF*	*rbcL*	*atpB-rbcL*	*psbA-trnH*	*trnTL*	*trnLF*	pseud*trnLF*	malate synthase	PHYC
R.E.Fr.												
Cremastosperma megalophyllum R.E.Fr.	Chatrou 268 (U)	Ecuador	AY743543	DQ018121	AY743520	—	DQ018239	DQ018146	AY743566	—	—	—
Cremastosperma megalophyllum R.E.Fr.	Chatrou 259 (U)	Ecuador	AY743545	DQ018122	AY743522	—	AY841451	DQ018147	AY743568	—	—	—
Cremastosperma microcarpum R.E.Fr.	Maas 8289 (U)	Peru	AY743539	—	AY743516	—	—	—	AY743562	—	—	—
Cremastosperma microcarpum R.E.Fr.	Chatrou 208 (U)	Peru	AY518874	DQ018120	AY319058	—	AY841452	DQ018145	AY319172	DQ018204	—	—
Cremastosperma monospermum (Rusby) R.E.Fr.	Pirie 4 (U)	Bolivia	AY743547	DQ018124	AY743524	—	AY841453	DQ018149	AY743570	DQ018205	—	—
Cremastosperma napoense Pirie	Neil 7649 (U)	Ecuador	DQ018265	DQ018127	DQ018224	—	DQ018242	DQ018152	DQ018177	DQ018208	—	—
Cremastosperma novogranatense R.E.Fr.	Devia 5335 (MO)	Colombia	AY743552	—	AY743529	—	—	DQ018156	AY743529	—	—	—
Cremastosperma oblongum R.E.Fr.	Maas 9148 (U)	Brazil	AY743549	DQ018126	AY743526	—	DQ018241	DQ018151	AY743572	DQ018207	—	—
Cremastosperma oblongum R.E.Fr.	Morawetz 25985 (U)	Peru	DQ018266	DQ018128	DQ018225	—	DQ018243	DQ018153	DQ018178	DQ018209	—	—
Cremastosperma oblongum R.E.Fr.	Pirie 7 (U)	Peru	DQ018273	DQ018137	DQ018232	—	DQ018253	DQ018165	DQ018185	DQ018215	—	—
Cremastosperma panamense Maas	Miller 947 (U)	Panama	AY743553	DQ018131	AY743530	—	DQ018246	DQ018158	AY743576	DQ018211	—	—
Cremastosperma pedunculatum (Diels) R.E.Fr.	Gentry 80904 (U)	Peru	DQ018269	—	DQ018228	—	DQ018248	DQ018161	DQ018181	—	—	—
Cremastosperma pedunculatum (Diels) R.E.Fr.	Gentry 45510 (U)	Peru	AY743561	DQ018135	AY743538	—	DQ018251	DQ018165	AY743584	DQ018215	—	—

Species	Collection	Country	*matK*	*ndhF*	*rbcL*	*atpB-rbcL*	*psbA-trnH*	*trnTL*	*trnLF*	pseud*trnLF*	malate synthase	PHYC
Cremastosperma pendulum (Ruiz & Pav.) R.E.Fr.	Morawetz 9888 (U)	Peru	AY743554	—	AY743531	—	DQ018247	DQ018159	AY743577	—	—	—
Cremastosperma pendulum (Ruiz & Pav.) R.E.Fr.	Graham 635 (U)	Peru	AY743555	DQ018132	AY743532	—	AY841454	DQ018160	AY743578	DQ018212	—	—
Cremastosperma pendulum (Ruiz & Pav.) R.E.Fr.	Pirie 33 (U)	Peru	DQ018274	DQ018138	DQ018233	—	DQ018254	DQ018168	DQ018186	DQ018218	—	—
Cremastosperma peruvianum	Rodríguez 1112 (U)	Peru	AY743557	—	AY743534	—	—	—	AY743580	—	—	—
Cremastosperma sp.	Barbosa 8008 (COL)	Colombia	MG649388	—	MG649389	—	MG680711	MG649390	MG649391	—	—	—
Cremastosperma aff. sp. B	Foster 3418 (US)	Peru	DQ018271	—	DQ018230	—	DQ018250	DQ018164	DQ018183	—	—	—
Cremastosperma stenophyllum Pirie	Knapp 6159 (COL)	Ecuador	MG680642	MG680643	MG680644	—	MG680645	MG680646	MG680647	MG680648	—	—
Cremastosperma venezuelanum Pirie	Steyermark 94314 (NY)	Venezuela	AY743559	DQ018134	AY743536	—	AY841457	DQ018163	AY743582	DQ018214	—	—
Cremastosperma westrae Pirie	Oliver 3681 (MO)	Panama	DQ018272	DQ018136	DQ018231	—	DQ018252	DQ018166	DQ018184	DQ018216	—	—
Cremastosperma yamayakatense Pirie	Vásquez 19055 (U)	Peru	DQ018267	—	DQ018226	—	DQ018244	DQ018155	DQ018187	—	—	—
Cremastosperma yamayacatense Pirie	Pirie 57 (U)	Peru	AY743558	DQ018139	AY743535	—	AY841458	DQ018169	AY743581	DQ018219	—	—
Cremastosperma yamayakatense Pirie	Pirie 80 (U)	Peru	DQ018275	DQ018141	DQ018234	—	DQ018255	DQ018171	DQ018179	—	—	—
Mosannona costaricensis (R.E.Fr.) Chatrou	Chatrou 90 (U)	Costa Rica	AY743503	AY841413	AY743510	AY841382	AY841479	—	AY743496	—	MG680650<br>MG680659	MG680683
Mosannona costaricensis (R.E.Fr.) Chatrou	Chatrou 91 (U)	Costa Rica	MG680704	—	MG680719	—	MG680712	—	MG680726	—	—	MG680684

Species	Collection	Country	*matK*	*ndhF*	*rbcL*	*atpB-rbcL*	*psbA-trnH*	*trnTL*	*trnLF*	pseud*trnLF*	malate synthase	PHYC
Mosannona depressa (Baill.) Chatrou subsp. *abscondita* Chatrou	Vester s.n. (U)	Mexico	MG680706	#	MG680721	MG680696	MG680714	—	MG680728	—	MG680652 MG680661	MG680685
Mosannona depressa (Baill.) Chatrou subsp. *depressa*	Calzada 1560 (U)	Mexico	MG680705	MG680668	MG680720	MG680695	MG680713	—	MG680727	—	MG680651 MG680660	MG680686
Mosannona discolor (R.E.Fr.) Chatrou	Jansen-Jacobs 6000 (U)	Surinam	AY743504	MG680669	AY743511	MG680697	AY841480	—	AY743497	—	MG680653 MG680662	MG680687
Mosannona garwoodii Chatrou & Welzenis	Garwood 3129 (U)	Panama	AY743505	MG680670	AY743512	MG680698	AY841481	—	AY743498	—	MG680654 MG680663	MG680688
Mosannona hypoglauca (Standl.) Chatrou	Herrera 923 (U)	Panama	MG680707	MG680671	MG680722	MG680699	MG680715	—	MG680729	—	—	MG680689
Mosannona pacifica Chatrou	Maas 8531 (U)	Ecuador	AY743506	MG680672	AY743513	MG680700	AY841482	—	AY743499	—	MG680655 MG680664	MG680690
Mosannona papillosa Chatrou	Pitman s.n (U)	Ecuador	AY743507	MG680673	AY743514	MG680701	AY841483	—	AY743500	—	MG680656 MG680665	MG680691
Mosannona parva Chatrou	Chatrou 23 (U)	Peru	MG680708	—	MG680723	—	MG680716	—	MG680730	—	—	MG680692
Mosannona raimondii (Diels) Chatrou	Morawetz 1828985 (U)	Peru	MG680709	—	MG680724	—	MG680717	—	MG680731	—	—	MG680693
Mosannona sp.	Chatrou 71 (U)	Costa Rica	AY743502	—	AY743509	—	AY841478	—	AY743495	—	—	MG680682
Mosannona vasquezii Chatrou	Chatrou 226 (U)	Peru	AY743508	MG680674	AY743515	MG680702	AY841484	—	AY743501	—	MG680657 MG680666	MG680694
Mosannona xanthochlora (Diels) Chatrou	Baker 6799 (U)	Ecuador	—	MG680675	—	—	MG680718	—	MG680732	—	—	—

# : Three small fragments of *ndhF* for *M. depressa* subsp. *abscondita* were included in the analyses, but were too small to be given GenBank accession numbers.
