# Supplementary material for: Parallel diversifications of Cremastosperma and Mosannona (Annonaceae), tropical rainforest trees tracking Neogene upheaval of South America
Source: R Soc Open Sci. 2018 Jan 31;5(1):171561. doi: 10.1098/rsos.171561 (PMC5792937; doi:10.1098/rsos.171561)
Supplement: Unique records for species distribution modelling [file rsos171561supp3.pdf]

Appendix 3

Mosannona	Area under the curve (AUC)	Number of unique records
costaricensis	1.000	4
depressa subsp. a*bscondita*	1.000	4
depressa depressa	0.988	145
discolor	0.994	6
hypoglauca	0.999	9
pacifica	1.000	5
papillosa	0.998	21
parva	0.995	7
raimondii	0.993	15
xanthochlora	0.998	6

Cremastosperma	Area under the curve (AUC)	Number of unique records
brevipes	0.986	14
cauliflorum	0.989	28
gracilipes	0.993	38
leiophyllum	0.994	14
longicuspe	0.996	5
macrocarpum	0.999	4
megalophyllum	0.994	44
microcarpum	0.979	21
monospermum	0.957	65
napoense	0.998	10
novogratense	0.999	5
oblongum	0.991	8
pacificum	0.999	5
panamense	0.999	11
pedunculatum	0.996	14
pendulum	0.994	5
peruvianum	0.998	6
yamyakatense	0.998	5
venezuelanum	0.999	4
spec B	0.993	13
