# Supplementary material for: Parallel diversifications of Cremastosperma and Mosannona (Annonaceae), tropical rainforest trees tracking Neogene upheaval of South America
Source: R Soc Open Sci. 2018 Jan 31;5(1):171561. doi: 10.1098/rsos.171561 (PMC5792937; doi:10.1098/rsos.171561)
Supplement: Independent climate and soil variables [file rsos171561supp4.pdf]

Appendix 3: The eight independent climate layers and ten independent soil variable layers used for species distribution modelling.

Bioclim variable	Explanation
BIO1	Annual Mean Temperature
BIO2	Mean Diurnal Range (Mean of monthly (max temp - min temp)
BIO4	Temperature Seasonality (standard deviation * 100)
BIO10	Mean Temperature of Warmest Quarter
BIO14	Precipitation of Driest Month
BIO16	Precipitation of Wettest Quarter
BIO18	Precipitation of Warmest Quarter
BIO19	Precipitation of Coldest Quarter

FAO variable	Explanation
BS_S	Base Saturation
CE_T	Cation Exchange Capacity Soil
CN_T	Carbon Nitrogen Ratio Top Soil
CP_S	Carbon Pool Soil
Depth	Soil Depth
Drain	Soil Drainage Class
NN_T	Nitrogen % Top Soil
SMAX	Soil Moisture capacity
Soil_prod	Soil production
Soil_text	Top Soil texture
